# Lights and Shadows

**Published:** 1897-01

**Authors:** 


### LIGHTS AND SHADOWS.

A Philadelphian, graduate of one of our leading veterinary schools, advertises information of a valuable character to owners of horses affected with fistulous withers. As yet he has not been known to give forth his discovery to the scientific veterinary world.

Some Orloff (Russian) trotting-mares are on their way to this country.

Every Eastern veterinary college seems to have received a full quota of veterinary students for the present year, and in 1897 there promises to be the largest number of students for many years, most of whom are equipping themselves for the veterinary sanitary police field of work.

The dismissals from the Bureau of Animal Industry at Omaha are said to have been ordered for "offensive partisanship" and the allowing of the same to interfere with official duties.

The first-year class of the Kansas City Veterinary College numbers some ten students, who are reported doing excellent work. The new graded course commenced the present session is being carried out and promises good results.

France continues, through her agents on this side, to be a large purchaser of American horses.

The Christmas number of the *Buffalo Horse World* presents to its readers an interesting galaxy of the noted speedy ones for 1896. It is an impressive review of the work of the trotters and pacers, and must add to the admiration of these pleasure-givers, health-invigorators, and money-winners to the great majority of all our people, who will never want to unlearn the lesson of love and admiration for the noblest animal on earth.

The noted Jersey bull, Pedro, lived to the ripe old age of nineteen years.

The Christmas number of the *Breeders' Gazette*, aside from its wealth of interesting reading matter, is one of the most handsomely illustrated numbers of any live-stock periodicals ever issued. The selection of noted animals and herds of live-stock of the highest types of breeding and of the greatest value is a great stimulus to the whole live-stock industry, and those engaged in this form of occupation owe a large debt of gratitude to the publishers of this valuable journal.

The half-hour talks by members of the several allied professions in special lines of work, given at the monthly meetings of the Keystone Veterinary Medical Association, are proving of the greatest interest and awakening many of the old members of the Association to renewed interest.

